# The influence of pro-longevity gene *Gclc* overexpression on the age-dependent changes in *Drosophila* transcriptome and biological functions

**DOI:** 10.1186/s12864-016-3356-0

**Published:** 2016-12-28

**Authors:** Alexey Moskalev, Mikhail Shaposhnikov, Ekaterina Proshkina, Alexey Belyi, Alexander Fedintsev, Svetlana Zhikrivetskaya, Zulfiya Guvatova, Asiya Sadritdinova, Anastasia Snezhkina, George Krasnov, Anna Kudryavtseva

**Affiliations:** 1 Engelhardt Institute of Molecular Biology, Russian Academy of Sciences, Moscow, Russia; 2 Institute of Biology of Komi Science Center of Ural Branch of RAS, Syktyvkar, Russia; 3 Moscow Institute of Physics and Technology, Dolgoprudny, Russia; 4 Syktyvkar State University, Syktyvkar, Russia

**Keywords:** Glutathione, Lifespan, Gene expression, Locomotor activity, Circadian rhythmicity, Fertility, *Drosophila melanogaster*

## Abstract

**Background:**

Transcriptional changes that contribute to the organism’s longevity and prevent the age-dependent decline of biological functions are not well understood. Here, we overexpressed pro-longevity gene encoding glutamate-cysteine ligase catalytic subunit (*Gclc*) and analyzed age-dependent changes in transcriptome that associated with the longevity, stress resistance, locomotor activity, circadian rhythmicity, and fertility.

**Results:**

Here we reproduced the life extension effect of neuronal overexpression of the *Gclc* gene and investigated its influence on the age-depended dynamics of transcriptome and biological functions such as fecundity, spontaneous locomotor activity and circadian rhythmicity, as well as on the resistance to oxidative, proteotoxic and osmotic stresses. It was shown that *Gclc* overexpression reduces locomotor activity in the young and middle ages compared to control flies. *Gclc* overexpression slowed down the age-dependent decline of locomotor activity and circadian rhythmicity, and resistance to stress treatments. *Gclc* level demonstrated associations with the expression of genes involved in a variety of cellular processes including Jak-STAT, MAPK, FOXO, Notch, mTOR, TGF-beta signaling pathways, translation, protein processing in endoplasmic reticulum, proteasomal degradation, glycolysis, oxidative phosphorylation, apoptosis, regulation of circadian rhythms, differentiation of neurons, synaptic plasticity and transmission.

**Conclusions:**

Our study revealed that *Gclc* overexpression induces transcriptional changes associated with the lifespan extension and uncovered pathways that may be associated with the age-dependent decline of biological functions.

**Electronic supplementary material:**

The online version of this article (doi:10.1186/s12864-016-3356-0) contains supplementary material, which is available to authorized users.

## Background

The stimulation of defense cellular systems leads to aging attenuation and lifespan extension in different organisms. For example, the enhancement of glutathione biosynthetic capability can determine longevity and delay aging. Previously, Dr. William Orr et al. demonstrated the pro-longevity role of glutamate-cysteine ligase (Gcl), which is a main catalyzer in the *de novo* glutathione synthesis [1]. Overexpression of genes encoding *Gcl* catalytic and modulatory subunits (*Gclc* and *Gclm*) was found to extend *Drosophila* lifespan and stimulate oxidative stress resistance without affecting the metabolic rate [1]. Additionally, previous studies have shown that the activity of *Gcl* and the amount of γ-glutamylcysteinylglycine (GSH), the protein that it synthesise, related to stress resistance levels [2, 3].

This paper aims to reveal pathways involved in the organism’s longevity and associated with the age-dependent decline of biological functions and stress resistance. We analyzed the effects of neuronal overexpression of *Gclc* on the lifespan, resistance to oxidative, proteotoxic and osmotic stresses, age-dependent dynamics of locomotor activity, fecundity, and transcriptomic changes. We reproduced the life extension effect of neuronal overexpression of the *Gclc* gene and demonstrated that *Gclc* overexpression slows down the age-dependent decline of locomotor activity and circadian rhythmicity without effect on fecundity. Transcriptome analysis revealed pathways that may contribute to the longevity and prevent the age-dependent decline of biological functions.

## Methods

### *Drosophila melanogaster* strains

#### *UAS-Gclc*

Carries an additional copy of the gene of catalytic subunit of glutamate-cysteine ligase (*Gclc*) under the control of UAS promoter on the chromosome 2. *Gclc* catalyzes the rate-limiting reaction in the *de novo* glutathione biosynthesis. Kindly provided by Dr. W.C. Orr (Southern Methodist University, Dallas, USA) [1].

#### *Appl-GAL4*

Driver line containing GAL4 selectively expressed in nervous system cells (#32040, Bloomington *Drosophila* Stock Center).

In order to match the genetic background of *UAS* and *GAL4* strains used in this study, flies were backcrossed into *w* ^*1118*^ (#3605, Bloomington *Drosophila* Stock Center, USA) for 6-8 times.

### Activation of overexpression

To activate the overexpression of the *Gclc* gene the *GAL4-UAS* system was used [4]. We used constitutively active neuronal driver *Appl-GAL4* that activate the *UAS-Gclc* overexpression in *Drosophila* heads [1]. *UAS-Gclc* flies from the parental line were used as a control.

### Lifespan analysis

The virgin females and males were used in the experiments. Animals were maintained in the Binder KBF720-ICH (Binder, Germany) climate chamber on the sugar-yeast medium at 25 °C in a 12 h light-12 h dark regime and at 60% relative humidity. Three-five *Drosophila* vials (Genesee Scientific, USA) containing 30 flies per vial were used in each experiment replication. Experiments were performed in 3 replicates. A total of 350-450 males and 350-450 females were analyzed. Animals were relocated to a fresh medium two times a week. Dead flies were counted daily. The median and maximum (as the age of 90% mortality) lifespan the were evaluated. The Mantel-Cox test was used to estimate the statistical differences in the median lifespan between control and experimental groups. The Wang-Allison test was used to compare the statistical differences in the maximum lifespan [5]. Statistical analysis was carried out using R (R core Team), version 2.15.1. The survival curves were plotted using STATISTICA, version 6.1 (StatSoft, USA).

### Sample collection and RNA isolation

Transcriptomic analysis was performed using control *UAS-Gclc* flies and flies with *Gclc* overexpression at the age of 1 (young), 4 (matured) and 6 weeks (old). Forty males and females heads were prepared separately for each experimental variant in 3 replicates. Fly transcriptome is strongly gender-dependent and one should not pool flies with different sex into one group. To reveal age-dependent and *Gclc*-induced transcriptomic changes, we applied multivariate generalized linear models (GLM) analysis. Total RNA was isolated from imago heads using QIAzol Lysis Reagent (Qiagen, Netherlands) with the isopropanol precipitation. The concentration of RNA was assessed using Qubit®2.0 Fluorometer (Invitrogen, USA) and NanoDrop® ND-1000 spectrophotometer (NanoDrop Technologies Inc., USA). The A260/A280 ratios of RNA samples were 1.8–2.0. The integrity of the isolated RNA (RIN) was measured with Bioanalyzer Agilent 2100 (Agilent Technologies, USA). RIN values for all samples didn’t be less than 8.0. All samples were treated with DNase I (Fermentas, Lithuania).

### Library preparation and sequencing of mRNA

Sample preparation and RNA sequencing were carried out by previously used protocol with modifications [6, 7]. The Illumina TruSeq™ RNA Sample Preparation Kit (Low-Throughput protocol) was used to prepare samples for mRNA sequencing libraries.

To purify poly-A containing mRNA molecules from total RNA samples (2.5 μg) the poly-T oligo-attached magnetic beads were used in two rounds of purification. During the second round of purification RNA was fragmented and primed for cDNA synthesis. Then the cDNA synthesis was performed using SuperScript Double-Stranded cDNA Synthesis Kit (Invitrogen, USA). cDNA was converted into the double-stranded (ds) cDNA. The ds cDNA was isolate from the second-strand reaction mix using Ampure XP beads.

The end-repair reaction was used to create blunt ends on the ds cDNA. To avoid the ligation of blunt ends during the adapter ligation reaction, a single ‘A’ nucleotide was added to the 3′ ends of them. The specific RNA Adapter Indexes supplied in the kit were ligated to cDNA fragments. The In-Line Control DNA was added to each enzymatic reaction. The PCR process (15 cycles) was used to selectively enrich DNA fragments with adapter molecules on both ends and to amplify the amount of DNA in the library, according to the manufacturer’s protocol.

The quantity of libraries was determined using the qPCR method by Rotor-Gene 6000 PCR System (Qiagen, USA) according to the manufacturer’s protocol. Primers matched sequences within adapters flanking an Illumina sequencing library. Before starting qPCR, a control template was selected to measure the libraries for quantification. The quality of libraries was defined using Agilent 2100 Bioanalyzer (Agilent, USA) according to the manufacturer’s protocol. The final product showed a band of approximately 260 base pairs.

cDNA libraries were normalized to 2nM, pooled together in equal volumes, and sequenced with 50 bp single-end reads on the HiSeq™2000 platform (Illumina, USA). Illumina HiSeq Analysis Software was used to obtain raw sequencing reads. The sequencing data were stored in FASTQ format. At least 25 million reads were obtained for each pool of flies.

### Sequencing data processing

Processing of transcriptomic data was performed using PPLine toolkit [8] including read preprocessing (trimmomatic), mapping (STAR) and counting (HTSeq-count). The further analysis was done with R programming language (R core Team). The edgeR package was used for analysis of differential expression [9]: we used Student *t*-test to compare two groups and generalized linear models (GLM) for complex comparisons in order to reveal genotype-, sex- and gender-associated transcriptomic changes on complete sampling (model ‘~ Age + Gender + Genotype). KEGG gene set enrichment analysis (GSEA) was performed using clusterProfiler [10]. To visualize altered KEGG pathways we modified pathview Bioconductor package [11] in order to take into account the absolute gene expression level, e.g. normalized read count per million (CPM) or FPKM. Aging is associated with global transcriptomic changes. Many KEGG nodes are mapped to multiple genes/proteins. For example, KEGG SdhA is related to both *FBgn0261439/SdhA* and *FBgn0036222/SdhAL*. SdhAL is a minor isoform of succinate dehydrogenase; it is overexpressed during the aging (LogFC_SdhAL_ = +5.2) and has the absolute expression level (CPM/FPKM) 100-fold lesser than its major isoform SdhA which is down-regulated in aged flies (LogFC_SdhA_ = -2.1). An original pathview package summarizes LogFC within each KEGG node, and the final LogFC for succinate dehydrogenase is +3.1, and this is not correct since minor *SdhAL* does not play any significant role here. To avoid this trouble, we have modified the pathview package to calculate the final LogFC as weighted sum of LogFC for each component: LogFC_final_ = (LogFC_SdhA_ · CPM_SdhA_ + LogFC_SdhAL_ · CPM_SdhAL_)/(CPM_SdhA_ + CPM_SdhAL_). This correction is critical when analyzing and visualizing transcriptomic changes affecting hundreds genes.

### Stress resistance dynamic analysis

To assess the changes in stress resistance of flies overexpressing the *Gclc* gene, 150 flies (30 flies per vial) were collected in each experimental variant at 10 different ages - 7, 14, 21, 28, 35, 42, 49, 56, 63, 70 days. Males and females were analyzed separately. Following stress treatments were used: oxidative (20 mM paraquat, 15 mM CuSO_4_), osmotic (400 mM NaCl) and proteotoxic stress (5 mM CdCl_2_). Flies were deprived of food and water for 3 h and were transferred into vials containing a filter paper moistened with 350 ml of the 5% sucrose solution with one of the substances. Two times a day, we counted the number of dead animals. Flies were transferred into new vials every two days. Flies were kept under stress until the end of life. The mean, median survival time, and the time of 90% mortality were calculated.

We determined the dependence of survival on age based on the Pearson correlation coefficient. Additionally, we evaluated the impact of age, gender, genotype and the nature of stress factors on the survival of flies by using Cox proportional hazards models. In the Cox regression analysis, we assumed that risk factors are the age of flies in which they were subjected to stress impact and their genotype. To determine the hazard ratio of death for each fly when exposed to stress, we compared contributions of sex and age specimens at different stresses for *UAS-Gclc* and *Appl-GAL4 > UAS-Gclc* flies. Next, we compared the risk for flies of each experimental group with the interaction of factors - gender and genotype. Thus, we determined how age, sex, and genotype affected the risk of death. Data analysis was performed using the STATISTICA software, version 6.1 (StatSoft, USA), OASIS [12], and R programming language (R Core Team).

### Analysis of fecundity and fertility

Before the analysis, females with and without overexpression of the *Gclc* gene were maintained with young wild-type *Canton-S* males for mating during 24 h. Mated females were put separately into the vials with a nutrient colored with activated carbon for egg-laying for 24 h. The number of eggs laid by females (fecundity) and the number of imago developed from the eggs by the 10-15th day after egg laying (fertility) were counted. Fecundity and fertility were estimated once a week. Experiment was performed in 2 replicates (a total of 100 females per experimental variant). To compare statistical significance between flies overexpressing the *Gclc* gene and flies without overexpression, *χ* ^*2*^ criterion was used.

### Locomotor activity analysis

The age-dependent dynamics of spontaneous locomotor activity was analyzed using *Drosophila* Locomotor Activity Monitor (Trikinetics, USA). Locomotor activity of 1, 4, and 6-week-old flies was recorded. The data were collected during 24 h and represented as average locomotor activity in 10 min bins or average total daily locomotor activity. The average locomotor activity was calculated between three vials each containing 10 flies.

### Circadian rhythmicity analysis

Locomotor activity of 5, 30, and 50-day-old males was recorded for 2 days in 12 h light : 12 h darkness conditions (LD), followed by 6 days in constant darkness (DD) using *Drosophila* Activity Monitor (Trikinetics, USA). For the analysis and visualization of circadian rhythmicity the ActogramJ software package [13] based on ImageJ Image Analysis Software [14] was used.

## Results

### Lifespan

**Fig. 1:**
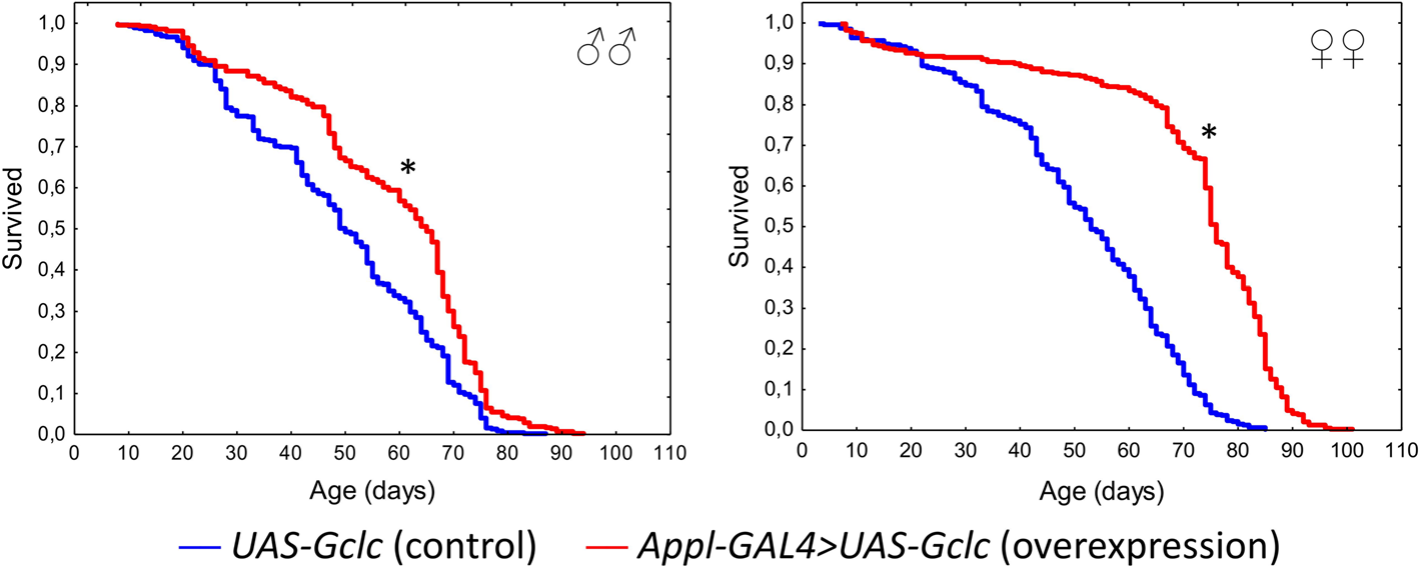
Influence of *Gclc* overexpression on lifespan (the results of 3 replicates are combined). *p < 0.001, Kolmogorov-Smirnov test

Overexpression of the *Gclc* gene led to the increase of median and maximum lifespan of *Drosophila* males and females compared with the control line *UAS-Gclc* (Table 1, Fig. 1). Thus, we reproduced the lifespan expending effect of *Gclc* overexpression, which was published earlier by Dr. William Orr et al. [1].

**Table 1:** Influence of *Gclc* overexpression on median and maximum lifespan (the results of 3 replicates are combined)

Genotype	Sex	M (days)	dM	Gehan-Wilcoxon test (*p*-value)	90% (days)	d90%	Wang-Allison test (*p*-value)	*n*
*UAS-Gclc* (control)	males	50			72			449
*Appl-GAL4 > UAS-Gclc (*overexpression)	males	65	+23.1%	*p* < 0.0001 (0)	76	+5.3%	*p* < 0.001 (0.00014)	419
*UAS-Gclc* (control)	females	53			72			464
*Appl-GAL4 > UAS-Gclc* (overexpression)	females	76	+30.3%	*p* < 0.0001 (0)	88	+18.2%	*p* < 0.0001 (0)	390

M - median lifespan, 90% - age of 90% mortality (maximum lifespan), dM - differences between median lifespan of control and *Gclc* overexpressed flies, d90% - differences between age of 90% mortality of control and *Gclc* overexpressed flies, *n* - number of flies

### Analysis of the transcriptome

We derived RNA-Seq expression profiles for 9400 genes (after eliminating low expression ones). We revealed the greatest transcriptomic changes during *Drosophila* aging: 3326 genes (*p* < 0.05) demonstrated expression associations with age (1885 of them passed FDR < 0.05 threshold), whereas alterations in 1442 (*p* < 0.05; 376 of 1442 have FDR < 0.05) we found to be sex-specific. Flies with enhanced *Gclc* activity showed lesser effect on gene expression profiles: only 188 genes (*p* < 0.05) were found to be associated with *Gclc* overexpression using the GLM model ‘~ Age + Gender + Genotype’ (*p* < 0.5; 28 of 188 have FDR < 0.05).

**Fig. 2:**
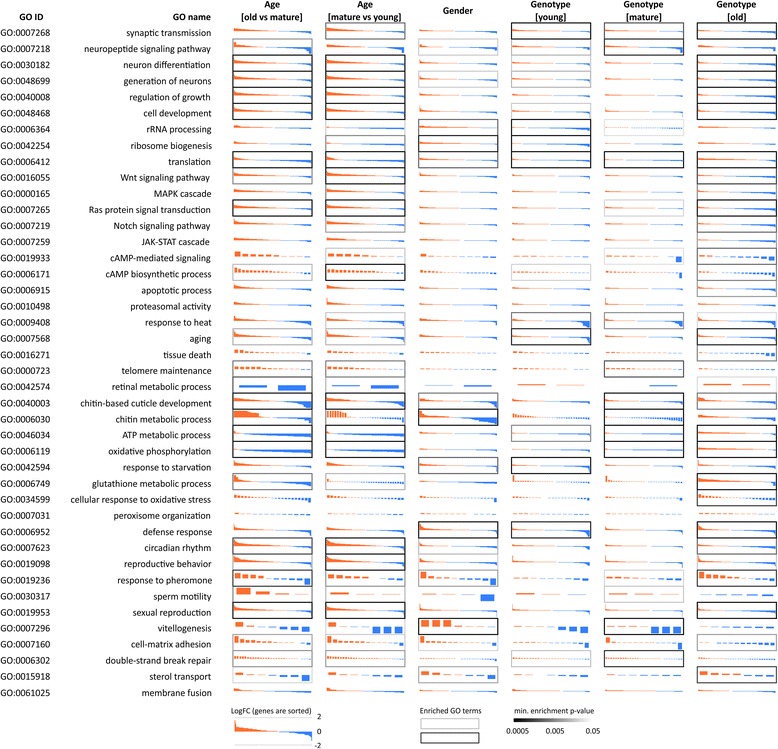
Differential expression profiles for genes participating in cellular pathways for different ages (young, mature, young), genders (males, females), and genotypes (*UAS-Gclc* and *Appl-GAL4 > UAS-Gclc* flies). Rectangle bars indicate minimal *p*-value for Gene Ontology GSEA

We performed GSEA based on GO annotation and pathway analysis based on KEGG data for differentially expressed genes that passed *p* < 0.05 threshold (Fig. 2, Additional file 1). We found the reduction of mitochondrial function, oxidative phosphorylation, ribosome biogenesis and translation as one of the hallmarks of aging. On the other hand, we found the age-dependent upregulation of apoptosis pathways, Wnt, mTOR/PI3K, FOXO pathways, circadian rhythm genes, enhanced cAMP signaling, overexpression of Myc and Notch.

### Stress resistance

**Fig. 3:**
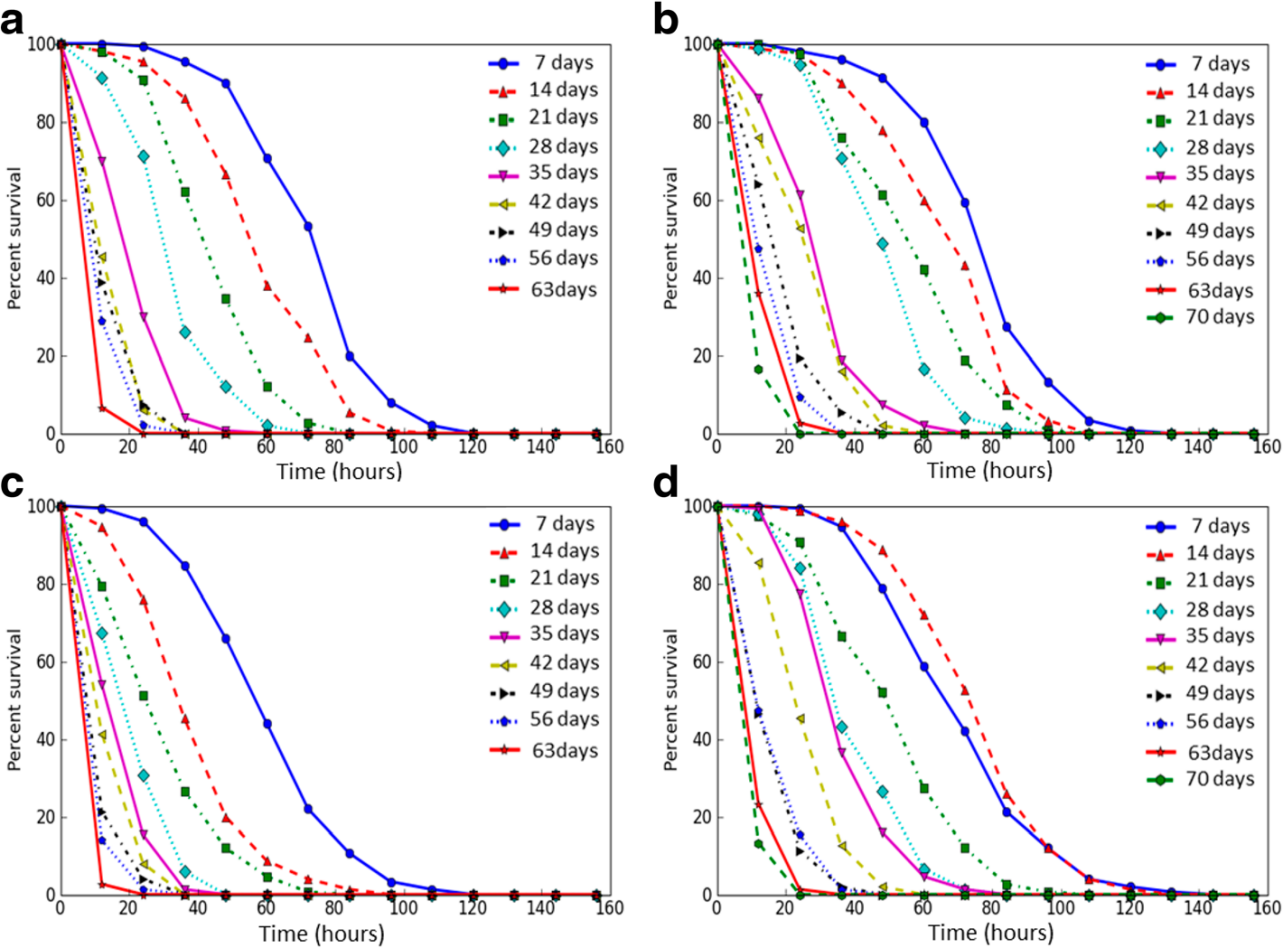
The survival curves of *Drosophila melanogaster* in different ages upon exposure to paraquat. **a** – *Appl-GAL4 > UAS-Gclc* males; **b** - *UAS-Gclc* males; **c** - *Appl-GAL4 > UAS-Gclc* females; **d** - *UAS-Gclc* females

**Fig. 4:**
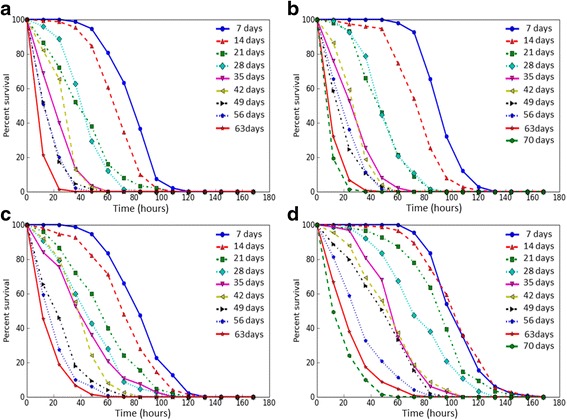
The survival curves of *Drosophila melanogaster* in different ages upon exposure to CuSO_4_. **a** – *Appl-GAL4 > UAS-Gclc* males; **b** - *UAS-Gclc* males; **c** - *Appl-GAL4 > UAS-Gclc* females; **d** - *UAS-Gclc* females

**Fig. 5:**
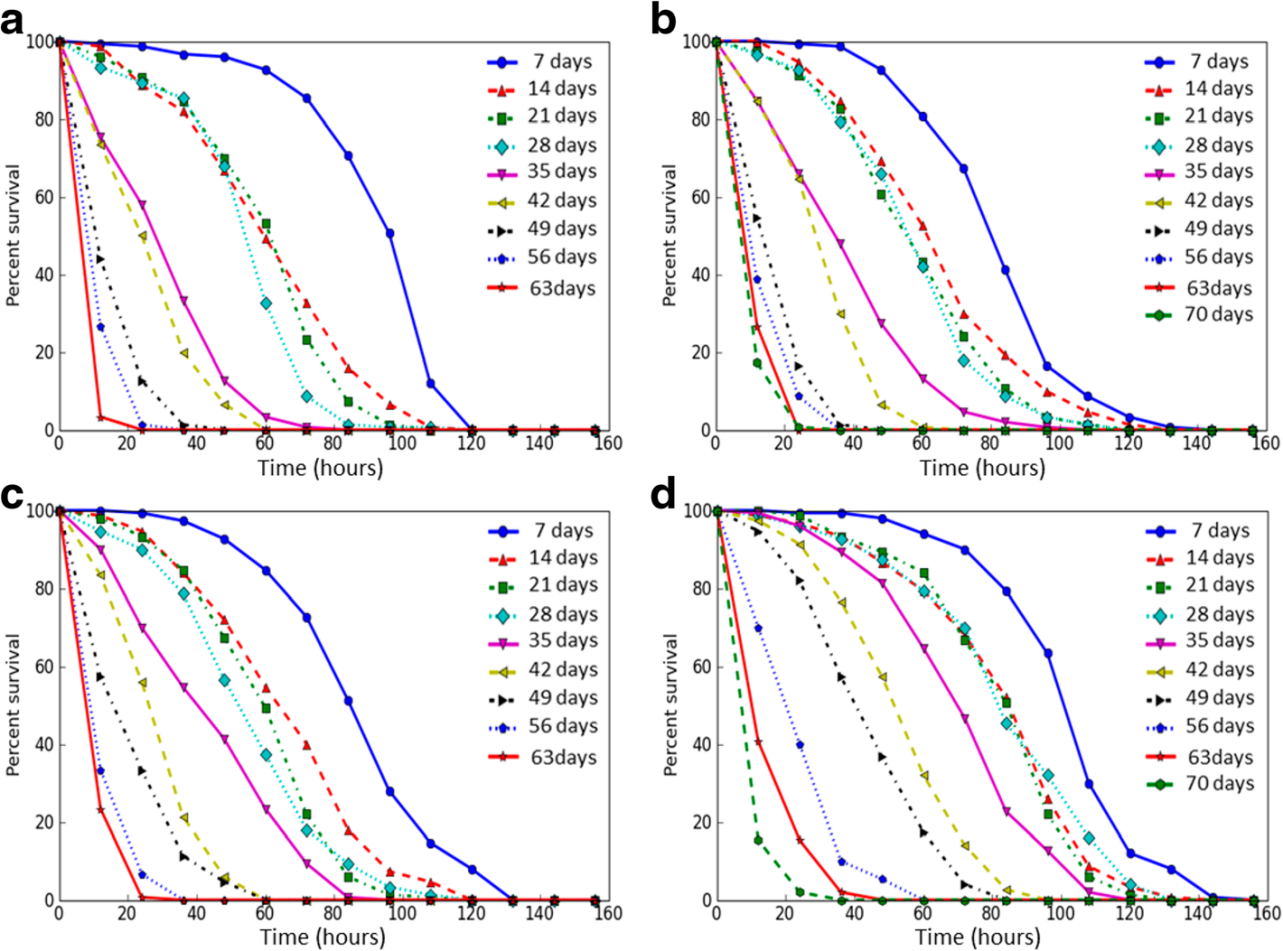
The survival curves of *Drosophila melanogaster* in different ages upon exposure to CdCl_2_. **a** – *Appl-GAL4 > UAS-Gclc* males; **b** - *UAS-Gclc* males; **c** - *Appl-GAL4 > UAS-Gclc* females; **d** - *UAS-Gclc* females

**Fig. 6:**
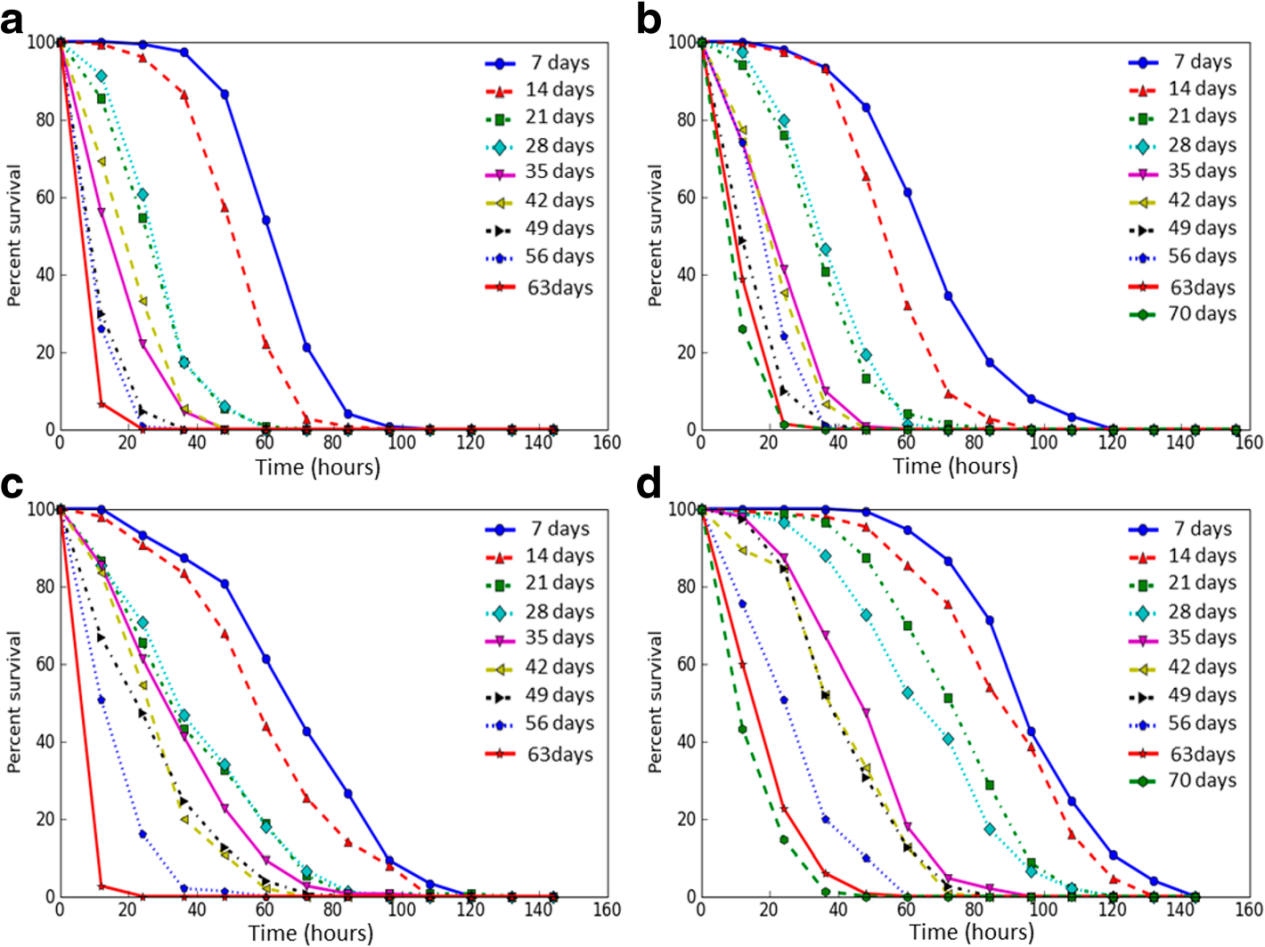
The survival curves of *Drosophila melanogaster* in different ages upon exposure to NaCl. **a** – *Appl-GAL4 > UAS-Gclc* males; **b** - *UAS-Gclc* males; **c** - *Appl-GAL4 > UAS-Gclc* females; **d** - *UAS-Gclc* females

We analyzed age-dependent changes of the resistance to different kinds of stress factors of flies with constitutive overexpression of the *Gclc* gene and the control line *UAS-Gclc*. Survival rates of flies under conditions of oxidative stress (paraquat, CuSO_4_), proteotoxic stress (CdCl_2_) and osmotic stress (NaCl) were estimated. Mean and median survival time, as well as the time of 90% mortality were decreased with age both in individuals with *Gclc* overexpression and in control flies under the treatment by all used stress factors (Table 2), that is discernible by the variation of survival curves (Figs. 3, 4, 5, and 6). Analysis using the Cox proportional hazard models also demonstrated that age is a risk factor in studied stress conditions for both genotypes (Table 3).

**Table 2:** The effect of age on survival of *Drosophila* after exposure to stresses

Stress and genotype of flies	Age (days)	Sex					
		Male			Female		
		X ± ∆m	M	90%	X ± ∆m	M	90%
Paraquat *UAS-Gclc*	7	76.6 ± 1.5	84	108	63.3 ± 1.7	60	96
	14	61.8 ± 1.6	60	84	42 ± 1.4	48	72
	21	48 ± 1.2	48	72	33 ± 1.4	36	60
	28	36.3 ± 1.1	36	60	24.5 ± 0.9	24	48
	35	24.6 ± 0.9	24	48	20.5 ± 0.7	24	48
	42	18.2 ± 0.6	24	48	17.9 ± 0.6	24	48
	49	17.5 ± 0.6	24	48	15 ± 0.5	24	48
	56	15.7 ± 0.5	24	48	13.8 ± 0.4	24	48
	63	12.8 ± 0.2	24	48	12.3 ± 0.2	24	48
Paraquat *Appl-GAL4 > UAS-Gclc*	7	80.3 ± 1.6	84	108	73.6 ± 1.8	72	108
	14	69.8 ± 1.6	72	96	78.2 ± 1.7	84	108
	21	60.4 ± 1.6	60	84	53.9 ± 1.6	60	84
	28	52.2 ± 1.3	48	72	43.2 ± 1.2	48	72
	35	33 ± 1.1	36	60	40.2 ± 1.1	48	72
	42	29.6 ± 1	36	60	29.4 ± 0.9	36	60
	49	22.6 ± 0.8	24	48	19.2 ± 0.7	24	48
	56	18.8 ± 0.6	24	48	19.7 ± 0.8	24	48
	63	16.6 ± 0.5	24	48	15 ± 0.5	24	48
	70	14 ± 0.4	24	48	13.6 ± 0.3	24	48
CuSO_4_ *UAS*-*Gclc*	7	82 ± 1.4	84	108	87.3 ± 1.7	84	120
	14	70.2 ± 1.4	72	96	76.2 ± 2	72	108
	21	45.4 ± 1.9	48	72	60.1 ± 2.1	60	96
	28	46.8 ± 1.3	48	72	50.1 ± 1.9	48	84
	35	27 ± 1.1	24	48	46.7 ± 2	48	84
	42	31.5 ± 1	36	60	43.4 ± 1.4	48	72
	49	21 ± 0.9	24	48	29.2 ± 1.4	24	48
	56	20.7 ± 0.8	24	48	24.4 ± 1.1	24	48
	63	14.7 ± 0.4	24	48	20.6 ± 0.9	24	48
CuSO_4_ *Appl-GAL4 > UAS-Gclc*	7	96.3 ± 1.2	96	120	107 ± 1.6	108	132
	14	78.3 ± 1.6	84	108	106 ± 1.9	108	132
	21	52.2 ± 1.4	48	72	93.9 ± 2.1	96	120
	28	53.8 ± 1.4	60	84	79.1 ± 2.2	72	120
	35	31.7 ± 1.3	36	60	60.6 ± 1.6	60	84
	42	32.2 ± 1	36	60	57.3 ± 1.9	60	84
	49	23.6 ± 1	24	48	52.2 ± 1.9	60	84
	56	25.7 ± 1	24	48	37.1 ± 1.6	36	72
	63	16.7 ± 0.6	24	48	28.3 ± 1.4	24	48
	70	14.5 ± 0.4	24	48	22.2 ± 1	24	48
CdCl_2_ *UAS-Gclc*	7	67.6 ± 1.1	72	96	72.6 ± 2	72	108
	14	55.8 ± 1.1	60	84	63.8 ± 1.9	60	96
	21	31.6 ± 1	36	60	42.6 ± 1.8	36	72
	28	33 ± 1	36	60	43.5 ± 1.8	36	72
	35	21.9 ± 0.9	24	48	38.9 ± 1.6	36	60
	42	25 ± 0.9	24	48	32.5 ± 1.2	36	60
	49	16.2 ± 0.6	24	48	30.7 ± 1.5	24	60
	56	15.2 ± 0.5	24	48	20.4 ± 0.8	24	48
	63	12.8 ± 0.2	24	48	12.3 ± 0.2	24	48
CdCl_2_ *Appl-GAL4 > UAS-Gclc*	7	71.9 ± 1.7	72	96	100.1 ± 1.7	96	132
	14	59.9 ± 1.2	60	84	91.8 ± 1.9	96	120
	21	39.5 ± 1.2	48	72	77 ± 1.7	84	108
	28	41.4 ± 1.1	48	72	69 ± 1.9	72	96
	35	27.1 ± 1	24	48	51 ± 1.4	48	72
	42	26.3 ± 0.8	36	60	44.7 ± 1.4	48	72
	49	19.2 ± 0.7	24	48	45.6 ± 1.3	48	72
	56	23.8 ± 0.7	24	48	30.7 ± 1.2	36	60
	63	16.8 ± 0.5	24	48	22.7 ± 0.9	24	48
	70	15.3 ± 0.5	24	48	19.1 ± 0.8	24	48
NaCl *UAS-Gclc*	7	96.2 ± 1.6	108	132	89.84	96	120
	14	65 ± 2	60	96	68.88	72	96
	21	63.2 ± 1.7	72	96	62.72	60	84
	28	57.6 ± 1.5	60	84	58.72	60	84
	35	34 ± 1.4	36	60	46.72	48	72
	42	30 ± 1.2	36	60	32	36	60
	49	19 ± 0.7	24	48	24.8	24	48
	56	15.4 ± 0.5	24	48	16.8	24	48
	63	12.4 ± 0.2	24	48	14.88	24	48
NaCl *Appl-GAL4 > UAS-Gclc*	7	85.1 ± 1.7	84	108	104.9 ± 1.8	108	132
	14	68 ± 2	72	96	85.6 ± 2.1	96	120
	21	61.8 ± 1.9	60	96	85.4 ± 1.8	96	120
	28	61 ± 1.8	60	84	86.6 ± 2.2	84	120
	35	41.6 ± 1.7	36	72	73.8 ± 1.9	72	108
	42	34.4 ± 1.1	36	60	56.6 ± 1.6	60	84
	49	20.7 ± 0.8	24	48	47 ± 1.5	48	72
	56	17.7 ± 0.6	24	48	27 ± 1.1	24	48
	63	15.2 ± 0.4	24	48	19 ± 0.8	24	48
	70	14.2 ± 0.4	24	48	14.1 ± 0.4	24	48

X ± ∆m - mean survival (h), M - median survival time (h), 90% - the time of 90% mortality (h)

**Table 3:** Cox regression calculation results for *Drosophila* after exposure to different stress factors

Genotype	Factor	Parameter	Stresses			
			Paraquat	CuSO_4_	CdCl_2_	NaCl
*Appl-GAL4 > UAS-Gclc*	Sex	HR	1.026	0.5152	0.4533	0.4533
		95% CI	0.9546-1.102	0.4783-0.555	0.4201-0.4891	0.4201-0.4891
*UAS-Gclc*	Age	HR	1.078	1.0681	1.0654	1.0824
		95% CI	1.0757-1.0802	1.0661-1.0701	1.0634-1.0674	1.0800-1.0848
	Sex	HR	1.5808	0.5105	0.4301	0.769
		95% CI	1.4650-1.7057	0.4724-0.5516	0.3975-0.4655	0.7128-0.8297
	Sex_male: *Appl-GAL4 > UAS-Gclc*	HR	0.5333	0.7105 95	0.5943	0.7561
		95% CI	0.4947-0.5749	0.6597-0.7651	0.5513-0.6407	0.7015-0.8150
	Sex_female: *Appl-GAL4 > UAS-Gclc*	HR	0.3558	0.3914	0.4064	0.3727
		95% CI	0.3296-0.3841	0.3625-0.4226	0.3769-0.4383	0.3451-0.4025

The first column shows the genotype compared flies in the analysis of the impact of these factors in the second column. HR - hazard ratio (the predicted risk of change when the value of the independent variable on the unit); 95% CI - a 95% confidence interval; Sex - a series of data showing the hazard regression for the flies with a certain genotype under the influence of each of the stress when factor is the individual’s sex; Age - a series of data showing the hazard regression for the flies with a certain genotype under the influence of each of the stress when factor is the individual’s age; Sex_male: *Appl-GAL4 > UAS-Gclc* a series of data showing the hazard ratio for flies with *UAS-Gclc* and *Appl-GAL4 > UAS-Gclc* genotypes when exposed to each of stresses, with the assessment of the impact of interacting factors (male sex, and genotype *Appl-GAL4 > UAS-Gclc*); Sex_female: *Appl-GAL4 > UAS-Gclc* a series of data showing the hazard ratio for flies with *UAS-Gclc* and *Appl-GAL4 > UAS-Gclc* genotypes when exposed to each of stresses, with the assessment of the impact of interacting factors (female sex, and genotype *Appl-GAL4 > UAS-Gclc*)

**Fig. 7:**
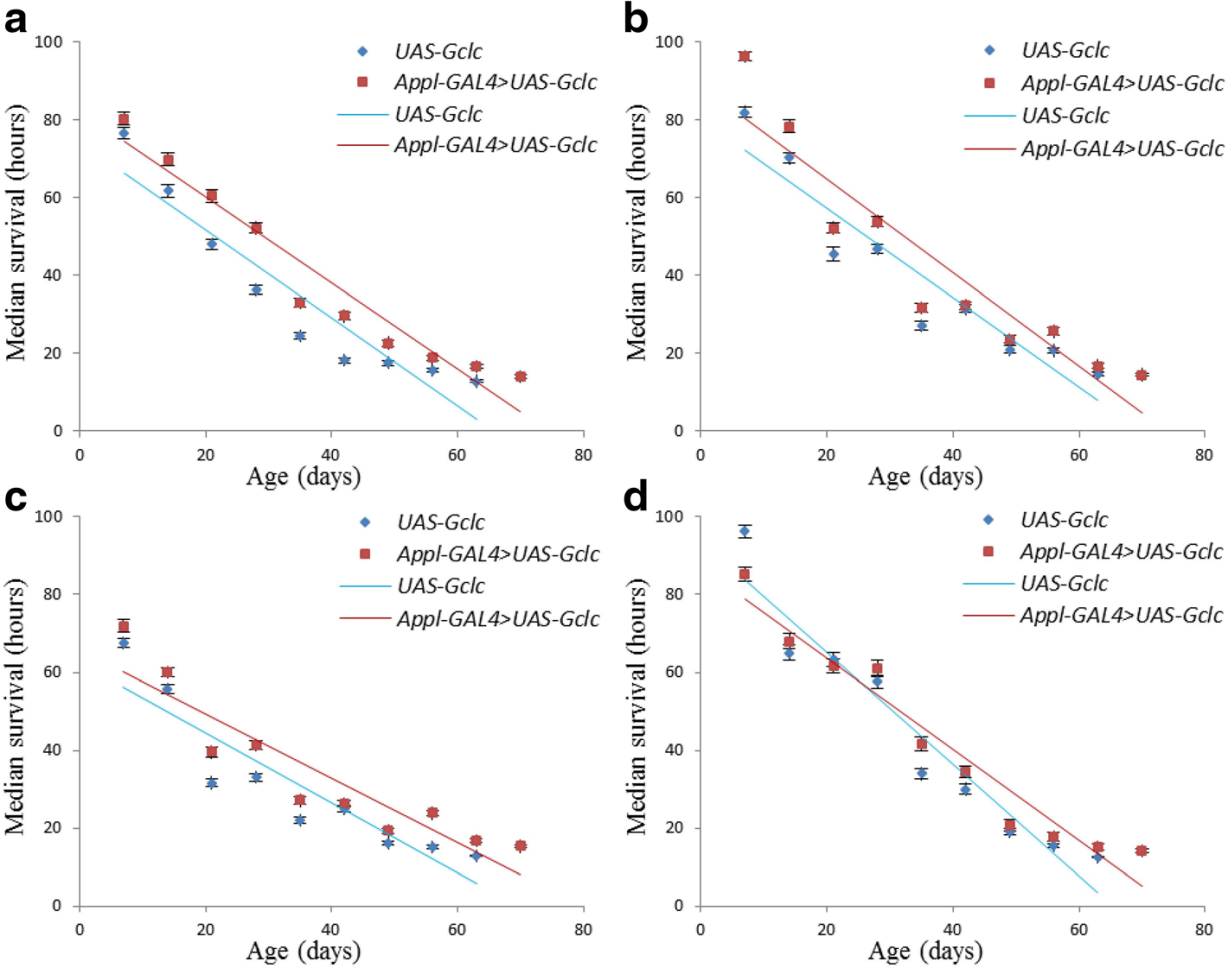
The dependence of survival on the age of *Appl-GAL4 > UAS-Gclc* and *UAS-Gclc* males. **a** - paraquat; **b** - CuSO_4_; **c** - CdCl_2_; **d** - NaCl. Color lines - linear regression

**Fig. 8:**
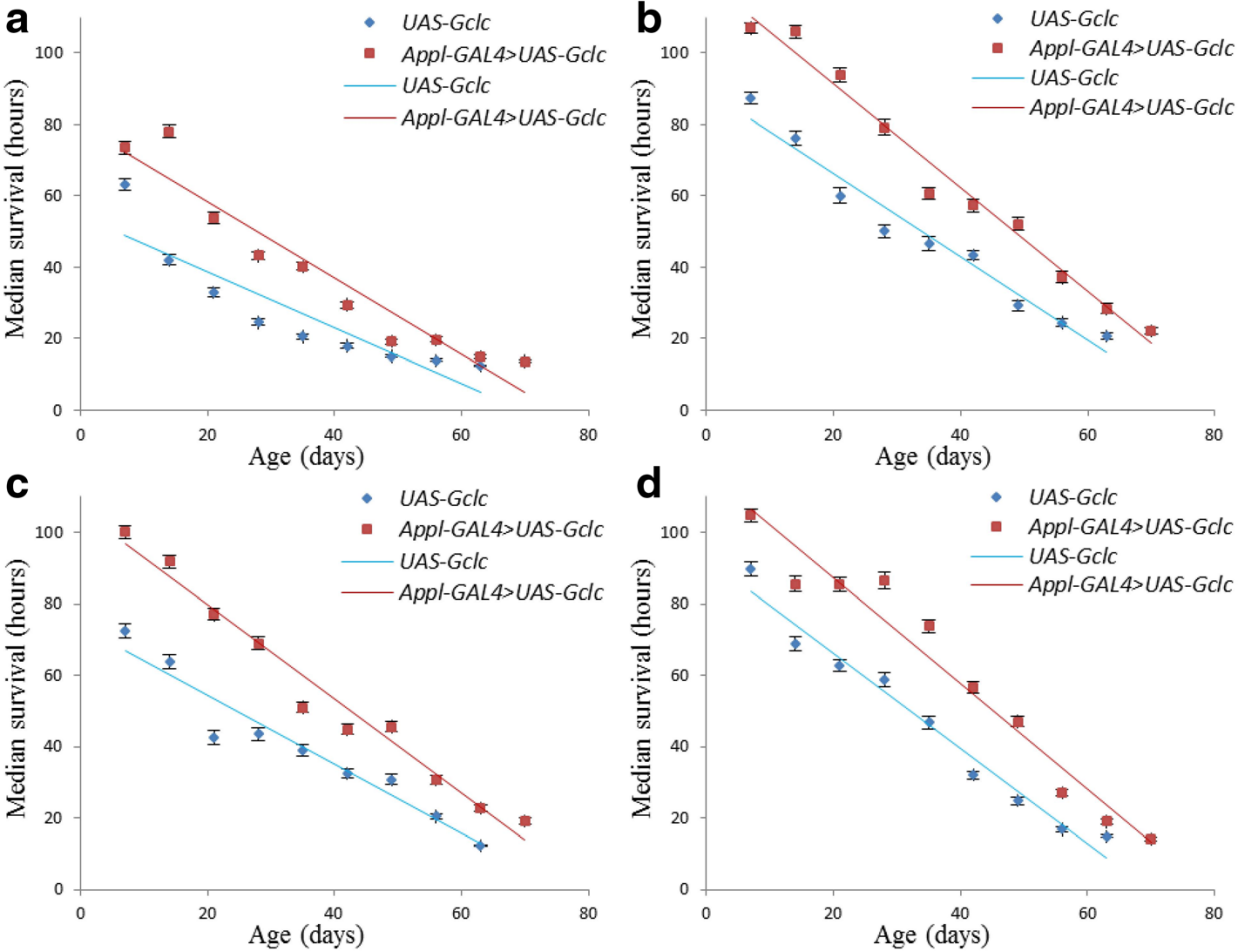
The dependence of survival on the age of *Appl-GAL4 > UAS-Gclc* and *UAS-Gclc* females. **a** - paraquat; **b** - CuSO_4_; **c** - CdCl_2_; **d** - NaCl. Color lines - linear regression

In order to compare the dynamics of survival of flies with and without overexpression of the *Gclc* gene, the correlation analysis was performed using the Pearson’s correlation coefficient. The correlation coefficient was calculated based on the mean survival time for all experimental variants at each age group (Table 4). A high negative correlation was revealed between survival and age in both males and females (Pearson’s correlation coefficient > 0.9; *p* < 0.001). This is also evident by the large angle of inclination of regression lines scatterplots (Figs. 7 and 8).

**Table 4:** Dependence of the average survival age of *Drosophila* after exposure to stresses

Variant of the experiment			*N*	Pearson *r*	*p*
Stress	Sex	Genotype			
Paraquat	Male	*UAS-Gclc*	9	−0.944	< 0.001
Paraquat	Male	*Appl-GAL4 > UAS-Gclc*	10	−0.968	< 0.001
CuSO_4_	Male	*UAS-Gclc*	9	−0.941	< 0.001
CuSO_4_	Male	*Appl-GAL4 > UAS-Gclc*	10	−0.934	< 0.001
CdCl_2_	Male	*UAS-Gclc*	9	−0.914	< 0.001
CdCl_2_	Male	*Appl-GAL4 > UAS-Gclc*	10	−0.922	< 0.001
NaCl	Male	*UAS-Gclc*	9	−0.964	< 0.001
NaCl	Male	*Appl-GAL4 > UAS-Gclc*	10	−0.974	< 0.001
Paraquat	Female	*UAS-Gclc*	9	−0.902	< 0.001
Paraquat	Female	*Appl-GAL4 > UAS-Gclc*	10	−0.958	< 0.001
CuSO_4_	Female	*UAS-Gclc*	9	−0.981	< 0.001
CuSO_4_	Female	*Appl-GAL4 > UAS-Gclc*	10	−0.990	< 0.001
CdCl_2_	Female	*UAS-Gclc*	9	−0.967	< 0.001
CdCl_2_	Female	*Appl-GAL4 > UAS-Gclc*	10	−0.986	< 0.001
NaCl	Female	*UAS-Gclc*	9	−0.986	< 0.001
NaCl	Female	*Appl-GAL4 > UAS-Gclc*	10	−0.978	< 0.001

*N* - the number of variables (median survival of flies at a certain age), Pearson *r* – the Pearson correlation coefficient, *p* – the level of significance of the correlation coefficient

To study age-dependent differences of stress resistance of *Appl-GAL4 > UAS-Gclc* flies and control *UAS-Gclc* flies, we calculated the value of the difference between the correlation coefficients (Table 5). In all cases, no significant differences in the age-dependent dynamics were found.

**Table 5:** The significance of differences of correlation coefficients for *Drosophila* after exposure to stresses

Stress	Sex	*p*
Paraquat	Male	0.30
CuSO_4_	Male	0.44
CdCl_2_	Male	0.46
NaCl	Male	0.40
Paraquat	Female	0.20
CuSO_4_	Female	0.27
CdCl_2_	Female	0.17
NaCl	Female	0.27

*p* – the significance level of the difference coefficients

Clouds variable in the scatter diagrams and linear regression in flies overexpressing *Gclc* in all variants except the males under exposure by NaCl are placed higher compared to the control line, thus it is possible to note a higher survival rate in different age groups as compared to the control line (Figs. 7 and 8). The linear regression of males’ survival under the influence of NaCl has similar values of the tilt angle and located close to each other. Thus, we can conclude that both flies with *Gclc* overexpression and without overexpression have similar levels of stress resistance.

Analysis using the Cox proportional hazards model demonstrated that under the influence of all examined stress males and females overexpressing *Gclc* have significantly lower risk of death compared with control line in all ages. Under the conditions of paraquat treatment, the stress tolerance of females overexpressing *Gclc* were not significantly differed from males, as well as *UAS-Gclc* females were less resistant to stress than males. In this study, overexpression of *Gclc* gene didn’t lead to significant changes in the dynamics of stress resistance. However, the previous work showed a higher resistance to oxidative stress in aged flies against a background of the increased activity of *Gclc*, and in young individuals stress resistance was on the level of control [1]. However, this effect was observed only in some embodiments of the experiment with a specific driver and dose of stress. Additionally, females with *UAS-Gclc* and *Appl-GAL4 > UAS-Gclc* genotypes were more resistant to stress than males, in spite of used treatment (Table 3).

### Fecundity and fertility

**Fig. 9:**
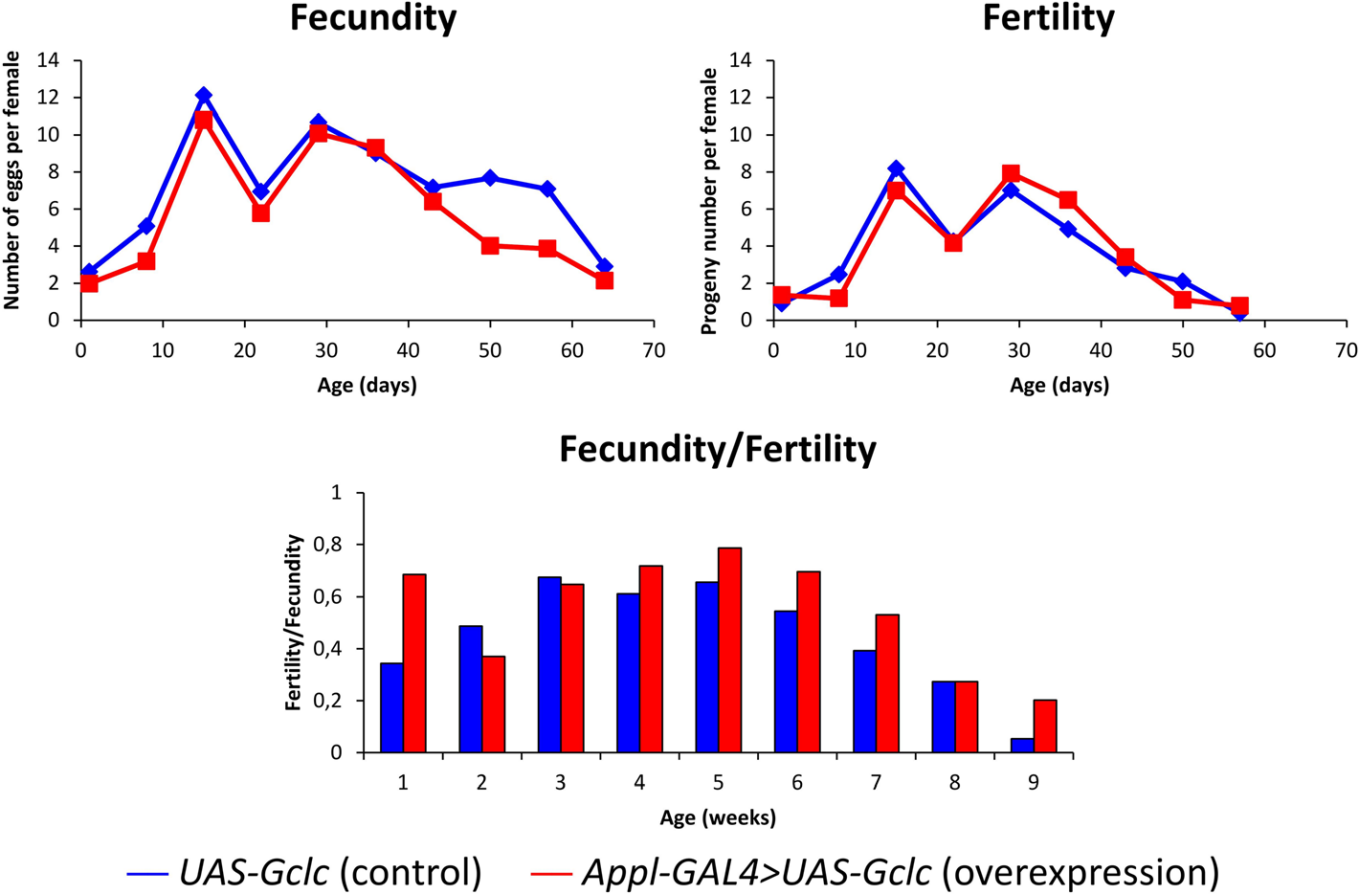
Influence of *Gclc* overexpression on age-dependent dynamics of fecundity and fertility

To estimate the age-dependent changes of fecundity and fertility, the number of eggs laid by females during 24 h was calculated every week during *Drosophila* lifetime, and the number of adult flies developed from these eggs was estimated after 10-15 days. We didn’t found statistically signifying differences of these parameters between females with *Gclc* overexpression and without overexpression (Fig. 9). However, the tendency to the increase of the percentage of progeny developed to imago was displayed in flies with increased *Gclc* expression (Fig. 9).

Surprisingly, we found the increase of the activity of many genes participating in the reproductive function during aging, except for genes involved in vitellogenesis which are primarily down-regulated both in aged flies and flies with *Gclc* overexpression. The fecundity and fertility analysis revealed the reduction of these parameters in aged flies and tendency to increase the percentage of imagoes developed from eggs of flies with *Gclc* overexpression compared with control.

### Locomotor activity

**Fig. 10:**
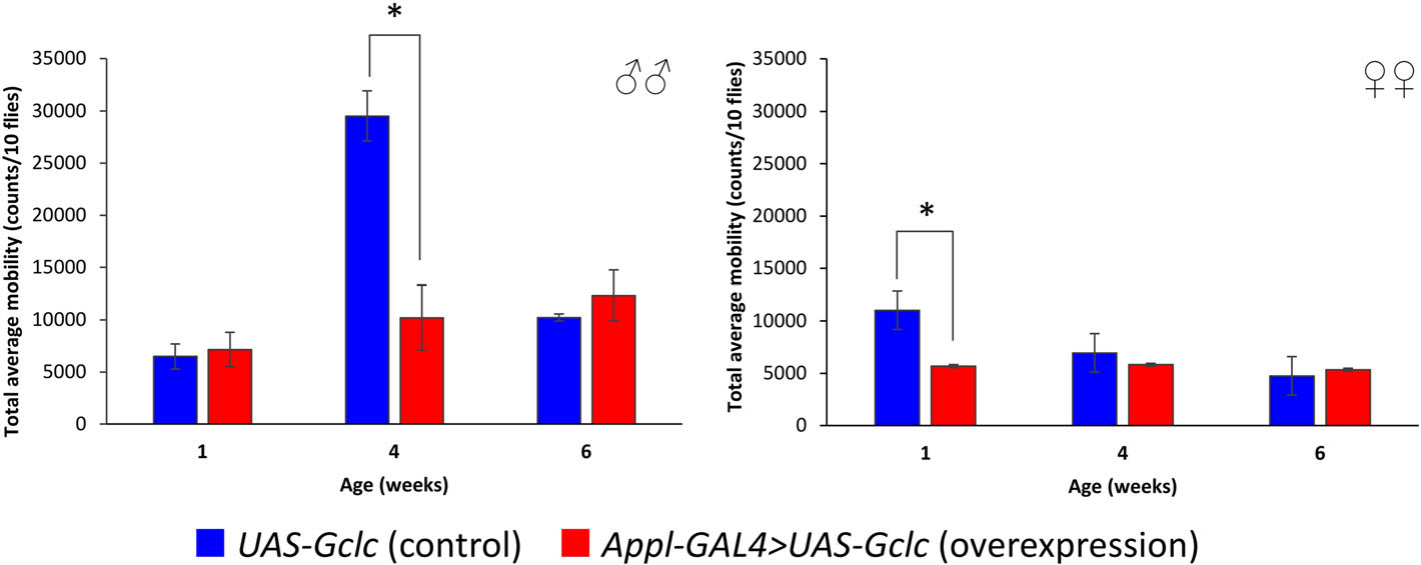
Influence of *Gclc* overexpression on age-dependent dynamics of total daily locomotor activity. *p < 0.05, *t*-Student test

The dynamics of age-dependent changes of locomotor activity was estimated in flies at the age of 1, 4, 6 weeks. It was found that *Gclc* overexpression decreased the total daily activity of 4-week-old males and 1-week-old females (Fig. 10). At the same time, the daily activity of flies with *Gclc* overexpression didn’t change during aging compared with control animals (Fig. 10).

**Fig. 11:**
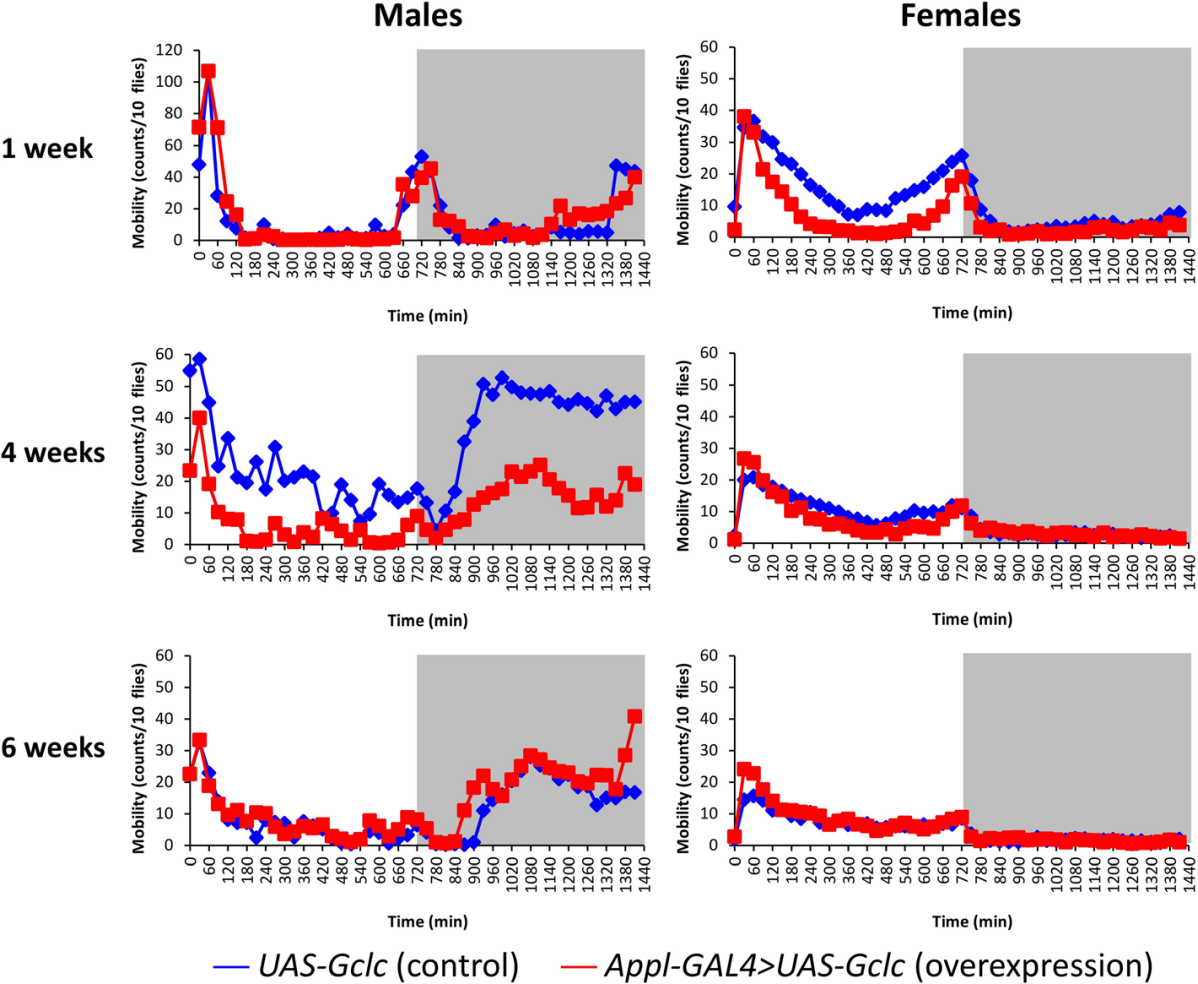
Effect of *Gclc* overexpression on age-dependent dynamics of average daily spontaneous locomotor activity. Vertical arrows indicate time of lights-off (ZT 12)

The analysis of average daily locomotor activity of flies at different ages educed that the decrease of locomotor activity level of 1-week-old females with *Gclc* overexpression is associated with the low activity level during light time (Fig. 11). At the same time, the decrease of locomotor activity of 4-week-old males with *Gclc* overexpression is conditioned by the low activity during dark time (Fig. 11). In the connection with alterations in daily activity of males and females, we investigated the influence of *Gclc* overexpression on the age-dependent dynamics of circadian rhythms.

### Circadian activity rhythms

**Fig. 12:**
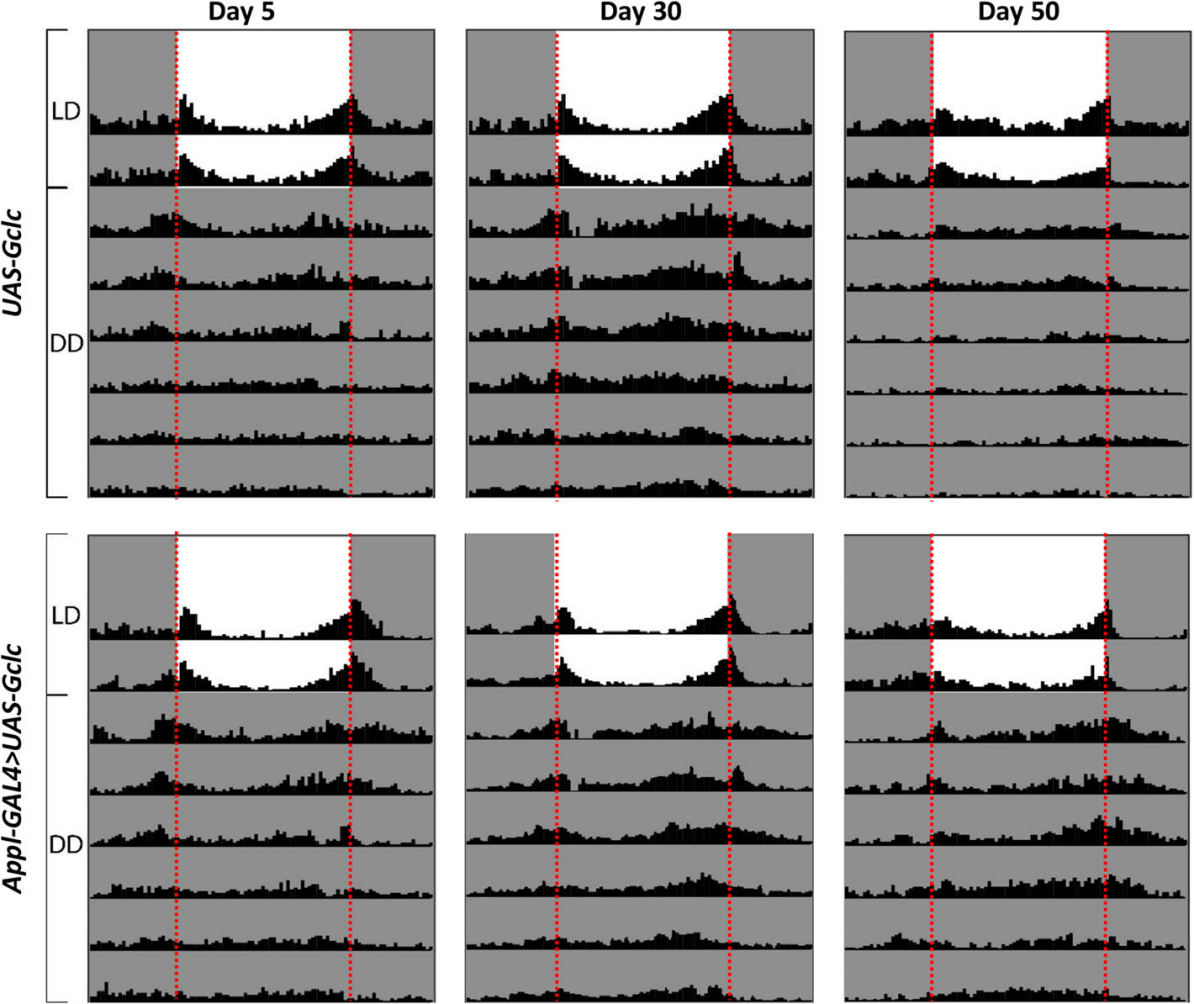
Influence of *Gclc* overexpression on age-dependent disruption of locomotor activity rhythms. Locomotor activity profiles of representative 5-, 30-, and 50-day-old males of the indicated genotypes. Flies of each age were monitored in LD (12:12) for 2-d, followed by 6-d in DD at 25 °C. Shaded areas represent periods of darkness. Vertical red dotted lines indicate time of lights-on (ZT/CT 0) and lights-off (ZT/CT 12)

For the estimation of age-dependent changes of circadian rhythms, we investigated daily changes of locomotor activity of males with *Gclc* overexpression and without overexpression at the age of 5-13, 30-38, and 50-58 days. At first, the locomotor activity was analyzed during 2 days under conditions of 12 h light : 12 h darkness (LD), then 6 days under conditions of 24 h darkness (DD). It was found that under LD conditions both control and *Gclc* overexpressing flies at the age of 5 and 30 days demonstrated morning and evening activity peaks before the switching on light, that is defined as zeitgeber time 0 (ZT 0), and switching off light at zeitgeber time 12 (ZT12) compared with 50-day-old flies (Fig. 12). At the age of 50 days both short-lived *UAS-Gclc* flies and long-lived *Appl-GAL4 > UAS-Gclc* flies had the smoothing of morning and evening activity peaks under LD conditions. The analysis of circadian rhythms of flies that were maintained under DD conditions demonstrated that 50-day-old flies overexpressing *Gclc* overexpression had more distinct morning and evening activity peaks compared with control flies (Fig. 12).

Additionally, we observed strong overexpression of most genes participating in regulation of circadian rhythms: *Per, Tim, Vri, Pdp, dCLK* [15, 16]. In old flies with and without *Gclc* overexpression, expression of *Per, Tim, Vri,* and *dCLK* was slightly down-regulated (Additional file 1). Obtained data demonstrated that *Gclc* overexpression led to slowing of age-dependent disruption of circadian activity.

## Discussion

We have found that the lifespan expending effect of overexpression of the *Gclc* gene in the nervous system protects from the age-dependent disruption of circadian rhythmicity and locomotor activity. This effect can be mediated by the activation of genes participating in regulation of circadian rhythms. Despite the lack of the effect of *Gclc* overexpression to fertility, we found increased activity of many genes participating in the reproductive function.

Overall, transcriptomic data demonstrated the activation of apoptosis pathways, Wnt, mTOR/PI3K, FOXO pathways, circadian rhythm genes, enhanced cAMP signaling, overexpression of Myc and Notch, as well as the decrease of mitochondrial function, oxidative phosphorylation, ribosome biogenesis, and translation as one of the hallmarks of aging. Previously, oxidative phosphorylation and related activities were found as only processes that are down-regulated in whole insect body according to the results of three previous transcriptomic studies of aging *Drosophila* [17–19]. Impairment of oxidative phosphorylation during aging is well known for other model organisms [20]. On the other hand, the stress response, including defense response and amino acid and purine metabolism, was found to be up-regulated during aging [21]. This is not supported with our data.

However, there is no great consensus between the different studies concerning other biological processes. Our results particularly contradict the data of Fabrice Girardot et al. [17]. Using microarray analysis, the authors have found that neuronal activity, especially response to light, is repressed during aging (3 days vs. 40 days old *Drosophila melanogaster*). In contrast, we found the predominant up-regulation of genes in the categories related to the neuronal function, excluding the neuropeptide signaling pathway, which was featured with the predominant down-regulation of expression. However, genes responsible for retinal metabolic processes were also found significantly repressed during aging. We confirmed that guanylyl cyclase alpha-subunit 99B (*Gycα99B*), which was previously found by Girardot et al. [17] as the most strongly associated with aging, demonstrates the perfect anti-correlation with aging according to our data (Spearman’s rank correlation coefficient *r* = -0.88, *p* < 10^−5^). In 2011, Pernille Sarup et al. found that longevity-selected lines of *Drosophila melanogaster* retain the young gene expression profile including differential expression of starvation response genes, down-regulation of genes participating in immune response and possibly, chronic inflammation [22].

Despite the fact that the changes of transcriptomic profiles introduced by activated *Gclc* are not so pronounced as the age- or gender-associated alterations, *Gclc*-associated changes are related to key processes that are involved in aging. Many genes that comprise ‘tissue death’ and ‘aging’ GO terms are up-regulated during aging of *Drosophila melanogaster*, but *Gclc* enhanced activity induced suppression of many genes related to these categories in old insects with *Gclc* overexpression (Fig. 2). *Gclc* inversed the aging-associated repression of cell respiration translation: it induced the upregulation of oxidative phosphorylation genes and ribosomal genes suggesting the overall increase of protein synthesis. The similar observation is true for cAMP pathways: overactivated *Gclc* downregulated the most of genes involved in cAMP signaling, activation of which is one of the aging hallmarks [23–25].

The analysis of KEGG pathways demonstrated *Gclc-*induced alterations in key cell signaling pathways that are involved in aging of a cell and organism. This effect was primarily observed for old individuals, but wasn’t detected for middle-aged and young imagoes. In aged flies with *Gclc* overexpression compared with control insects, we observed: decrease of many genes comprising MAPK pathway associated with aging and stress [26, 27], aging-associated mTOR/PI3K pathway [28], and FOXO. However, the latter one is thought to be a contributor to longevity, stress resistance and tumor suppression [29].

The expression of eight genes, *SMC2* (*Structural maintenance of chromosomes 2*), *w* (*white*), *CG4293, Gclc, Cyp4p2* (*cytochrome P450 4p2*), *Ipk1* (*Inositol-pentakisphosphate 2-kinase*), *CG8157, CR45457* demonstrated strikingly strong association with *Gclc* overexpression in all of the groups: young/mature/old or males/females. We observed 1.5-4-fold decrease of various Turandot family members (*TotC, TotM, TotX, TotA*) in *Drosophila melanogaster* with *Gclc* overactivation of all ages (young, mature and old). Members of this family are induced in the response to various stresses including starvation, toxic substances, irradiation, infectious agents [6, 30]. Most likely, down-regulation of the Turandot family is a result of the overall reduction in stress load (including oxidative stress). This hypothesis is indirectly supported by the predominant decrease of many genes of category ‘defense response’ as well as heat shock proteins responsible for protein refolding (‘response to heat’ terms). The expression of *Tot* genes is regulated by JAK-STAT and MAPK pathways [31]. The elements of these two pathways were significantly down-regulated in flies with *Gclc* overexpression, according to the KEGG pathway analysis (Additional file 1).

Analysis of the influence of age on the survival of *Drosophila melanogaster* with constitutive overexpression of the *Gclc* gene and control flies in oxidative, proteotoxic and osmotic stress conditions showed that both genotypes characterized by the negative correlation between age and stress tolerance. The gradual deterioration of the ability to respond to stress with age may be a general aging mechanism for different organisms [32–34]. For *Drosophila melanogaster* and a number of other species it has been shown that the increase in lifespan is accompanied by the impairment of the resistance to various stress factors [35–37]. On the other hand, accelerated aging is often accompanied by a decrease in stress tolerance [38]. Additionally, our study found no significant difference in the dynamics of stress tolerance at different ages throughout the entire range of the studied stresses, as well as in different sexes. This supports the idea that there is an infringement of physiological functions and homeostasis associated with aging, such changes are common to the different sexes and affect a wide range of stress response mechanisms [39].

Overall, the survival in the conditions of oxidative, proteotoxic and osmotic stresses of flies overexpressing the *Gclc* gene is significantly higher compared with control flies. These stresses are directly or indirectly lead to increased oxidative stress in the body. Therefore elevated levels of GSH has a positive effect on the survival of flies [40, 41]. Aging reduces the content of GSH in cells, as well as disturbs maintaining a certain level of GSH at oxidative stress conditions [42]. One of mechanisms of the occurrence of these effects may be the violation of GSH synthesis mechanisms and its affinity for the substrate [43]. Thus, *Gclc* overexpression increases the organism ability to resist stress at all ages, but it is not able to slow down the dynamics of the reduction of stress resistance.

## Conclusion

Our study revealed that *Gclc* overexpression induces transcriptional changes associated with lifespan extension and uncovered pathways that may be associated with the age-dependent decline of biological functions. The *Gclc* level demonstrated associations with expression of genes involved in a variety of cellular processed including Jak-STAT, MAPK, FOXO, Notch, mTOR, TGF-beta signaling pathways, translation, protein processing in endoplasmic reticulum, proteasomal degradation, glycolysis, oxidative phosphorylation, apoptosis, regulation of circadian rhythms, differentiation of neurons, synaptic plasticity and transmission.

## Additional file

Additional file 1: Upregulated and downregulated pathways changing activity during aging of flies with overexpression of the *Gclc* gene and without overexpression based on KEGG data. This figure shows both genes with statistically significant differential expression (*p* < 0.05) and genes that only demonstrate up/down-regulation trends (statistically insignificant; almost all of them have low absolute LogFC values and are marked with light shades). (PDF 1137 kb)

## Abbreviations

Gclc: Glutamate-cysteine ligase catalytic subunit
