# Supplementary material for: The influence of pro-longevity gene Gclc overexpression on the age-dependent changes in Drosophila transcriptome and biological functions
Source: BMC Genomics. 2016 Dec 28;17(Suppl 14):1046. doi: 10.1186/s12864-016-3356-0 (PMC5249042; doi:10.1186/s12864-016-3356-0)
Supplement: Additional file 1: — Upregulated and downregulated pathways changing activity during aging of flies with overexpression of the Gclc gene and without overexpression based on KEGG data. This figure shows both genes with statistically significant differential expression (p < 0.05) and genes that only demonstrate up/down-regulation trends (statistically insignificant; almost all of them have low absolute LogFC values and are marked with light shades). (PDF 1137 kb) [file 12864_2016_3356_MOESM1_ESM.pdf]

Additional file 1

Jak-STAT signaling pathway

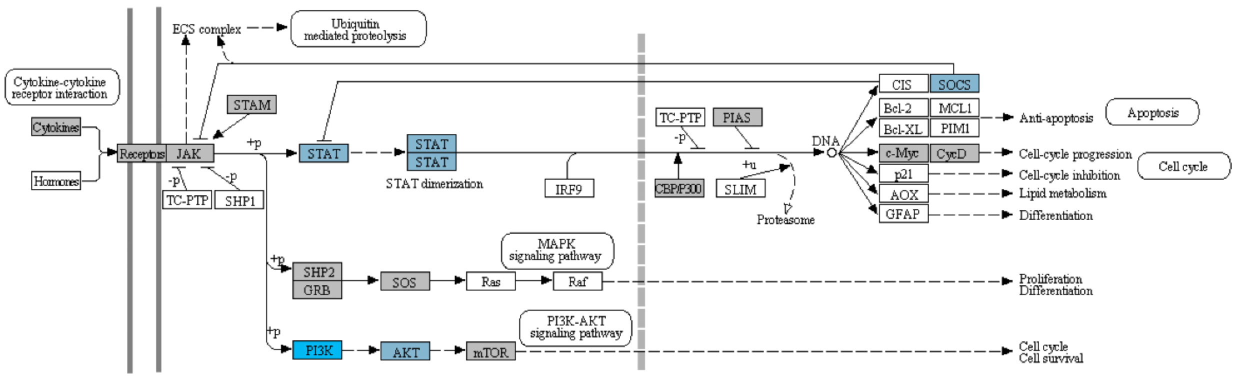

Circadian rhythm

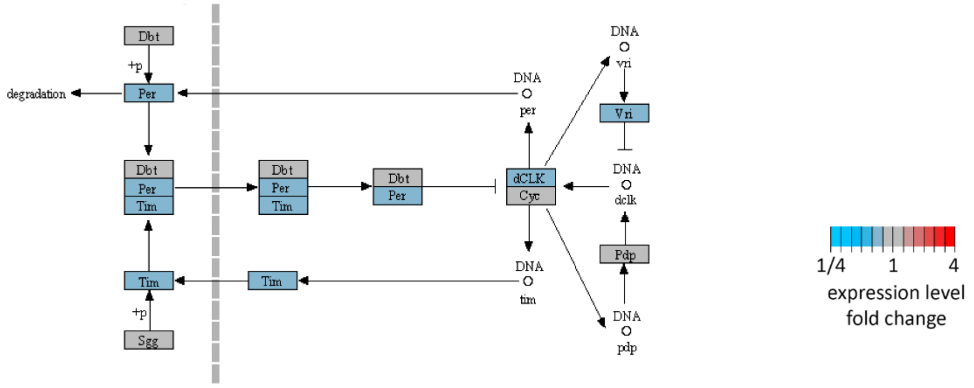

MAPK signaling pathway

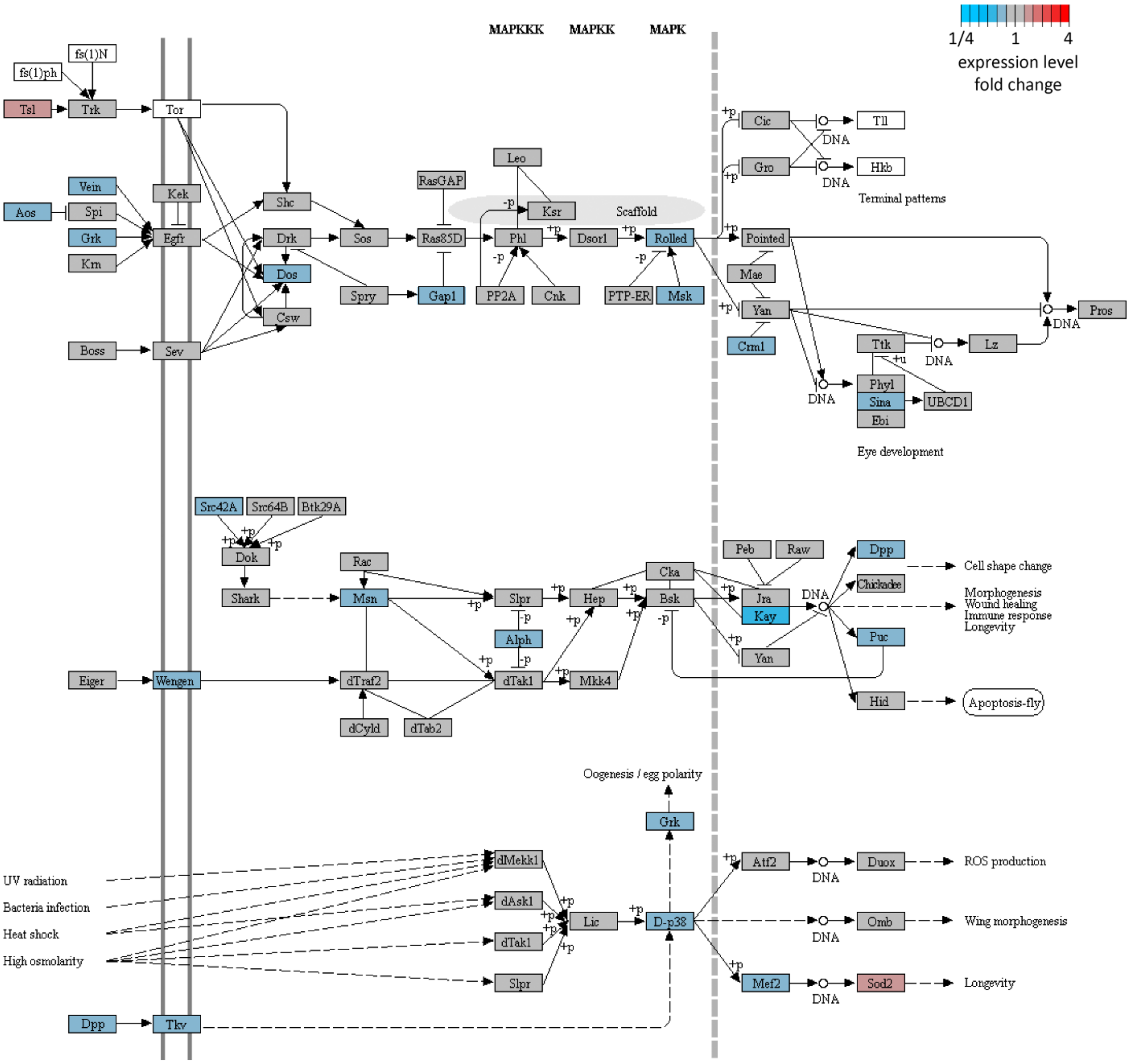

FOXO signaling pathway

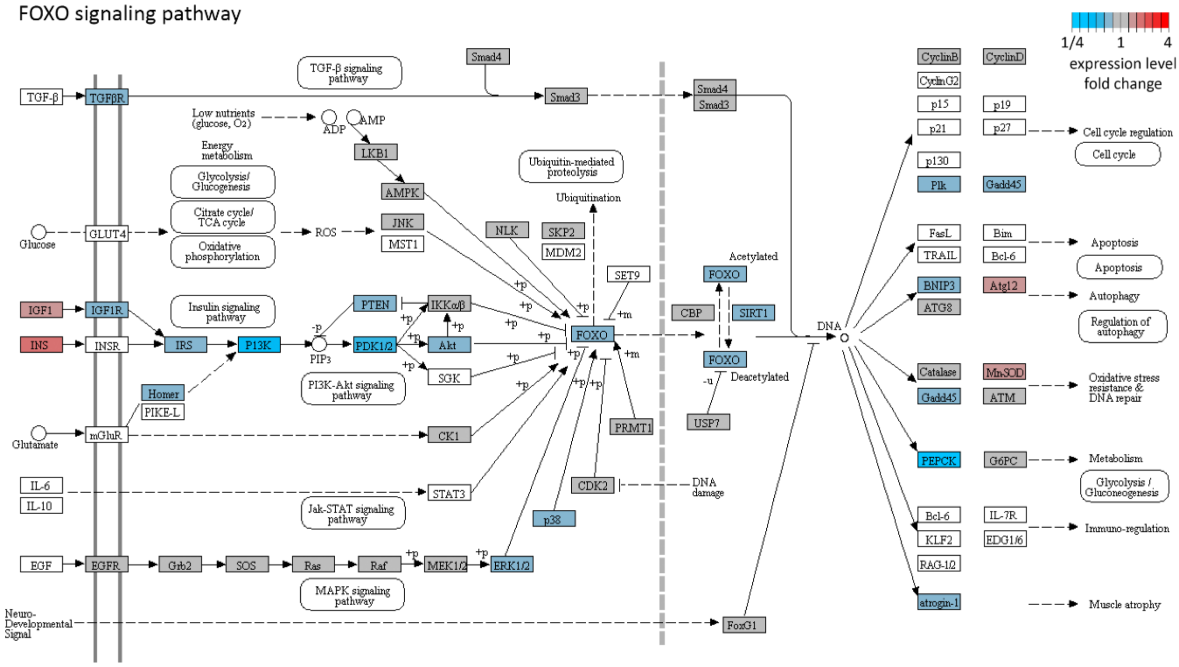

Ribosome biogenesis

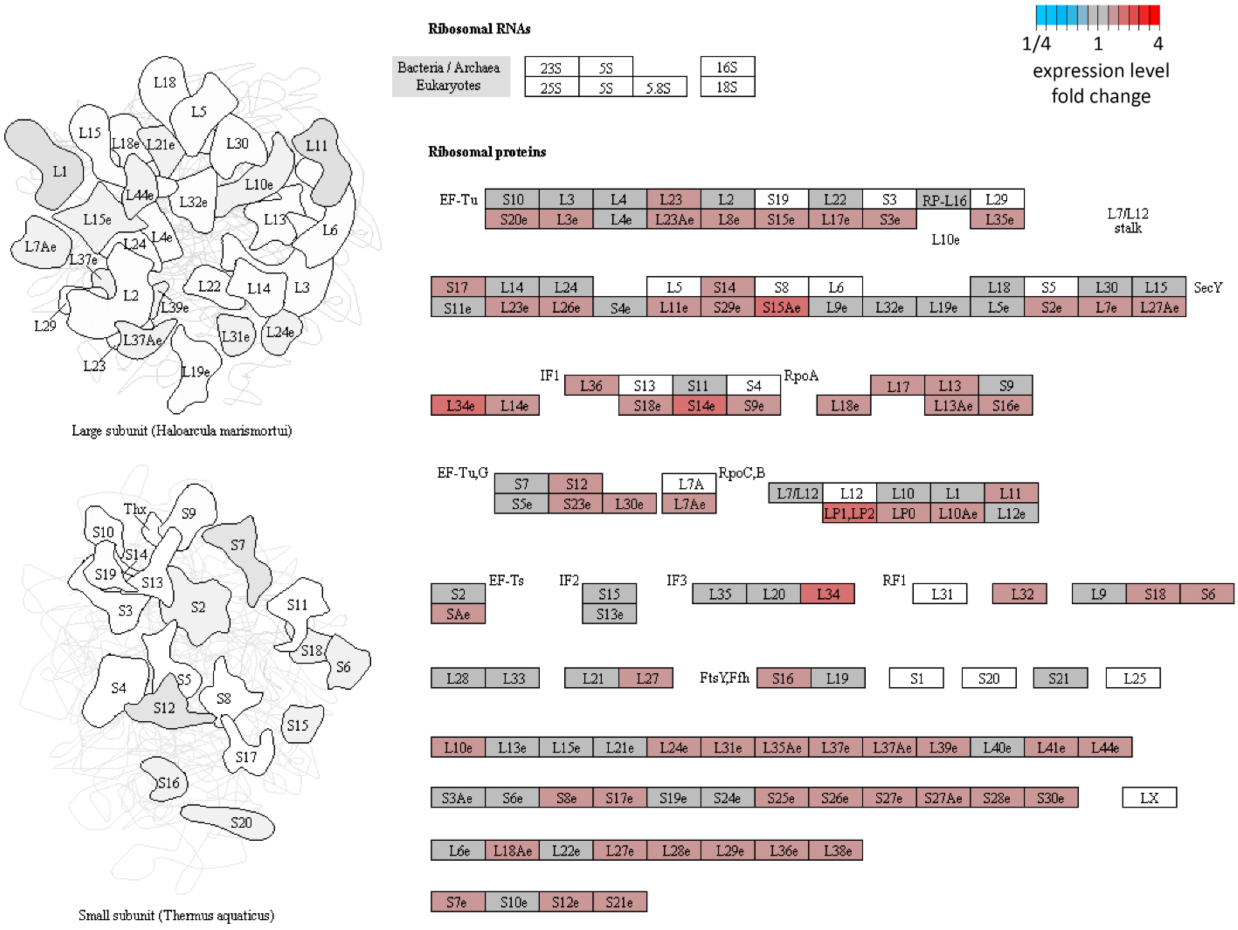

Oxidative phosphorylation

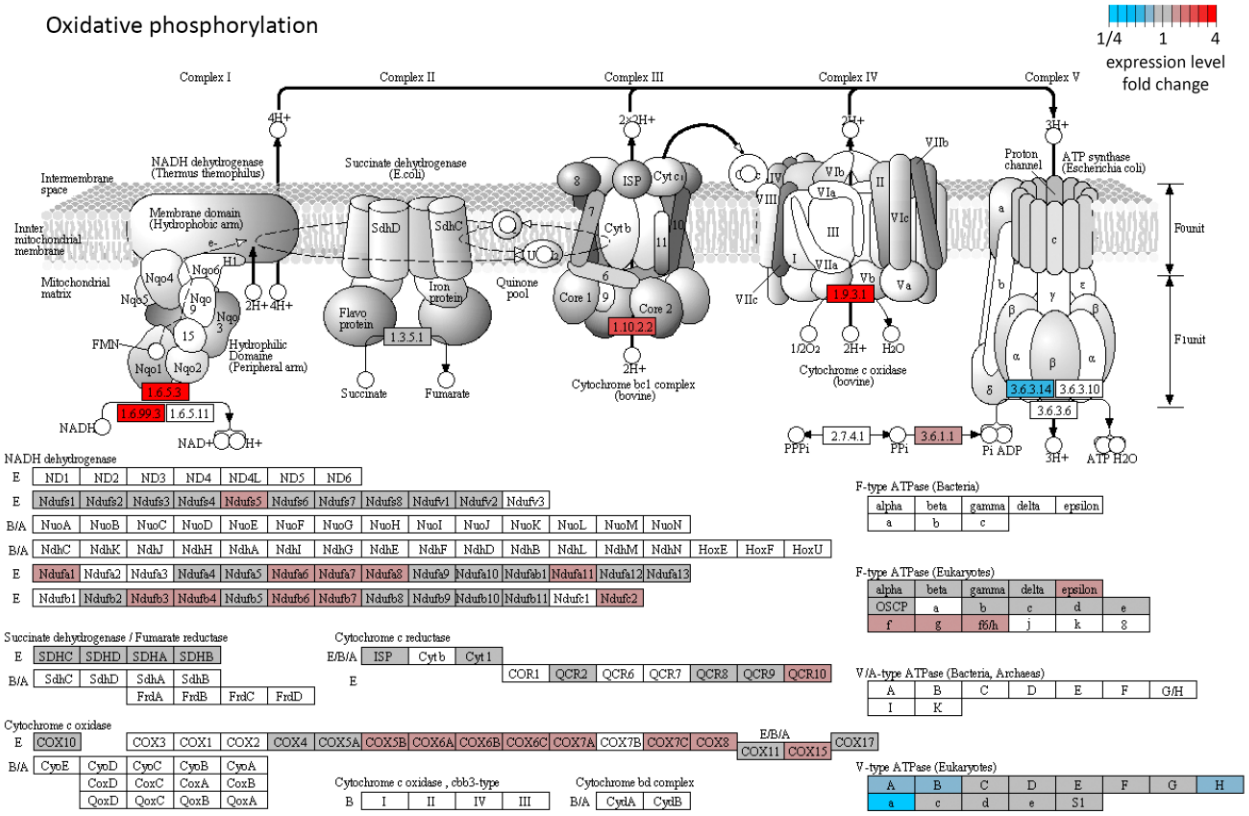

Additional file 1. Upregulated and downregulated pathways changing activity during aging of flies with overexpression of the *Gclc* gene and without overexpression based on KEGG data. This figure shows both genes with statistically significant differential expression ($p < 0.05$) and genes that only demonstrate up/down-regulation trends (statistically insignificant; almost all of them have low absolute LogFC values and are marked with light shades).
